# Improvement of blood inflammatory marker levels in patients with hypothyroidism under levothyroxine treatment

**DOI:** 10.1186/s12902-015-0032-3

**Published:** 2015-06-23

**Authors:** Roseane C. Marchiori, Luiz A. F. Pereira, Alexandre A. Naujorks, Diego L. Rovaris, Daiane F. Meinerz, Marta M. M. F. Duarte, João B. T. Rocha

**Affiliations:** Departamento de Clinica Médica, Centro de Ciencias da Saude, Universidade Federal de Santa Maria, Av. Roraima n° 1000, Cidade Universitaria, Camobi, Santa Maria, RS Brazil; Serviço de Metodos Graficos, Hospital Universitario de Santa Maria, Universidade Federal de Santa Maria, Av. Roraima n° 1000, Cidade Universitaria, Camobi, Santa Maria, RS Brazil; Departamento de Genetica, Instituto de Biociencias, Universidade Federal do Rio Grande do Sul, Av. Bento Gonçalves, 9500 Porto Alegre, Brazil; Departamento de Bioquímica e Biologia Molecular, Centro de Ciencias Naturais e Exatas, Universidade Federal de Santa Maria, Av. Roraima n° 1000, Cidade Universitaria, Camobi, Santa Maria, RS Brazil; Ciencias da Saude, Universidade Luterana do Brasil (ULBRA), campus Santa Maria, BR 287, Km 252, Trevo Maneco Pedroso, Boca do Monte, Santa Maria, RS Brazil Cx. Postal 21834

**Keywords:** Inflammation, Oxidative stress, Overt hypothyroidism, Subclinical hypothyroidism, Hashimoto’s thyroiditis, Levothyroxine, Atherosclerosis

## Abstract

**Background:**

There are several specific inflammatory and oxidative correlates among patients with hypothyroidism, but most studies are cross-sectional and do not evaluate the change in parameters during the treatment. The aim of this study was to investigate the effect of levothyroxine replacement therapy on biomarkers of oxidative stress (OS) and systemic inflammation in patients with hypothyroidism.

**Methods:**

In this prospective open-label study, 17 patients with recently diagnosed primary hypothyroidism due to Hashimoto’s thyroiditis who were not taking levothyroxine were included. The following parameters were measured before and at 6 and 12 months of levothyroxine treatment with an average dose of 1.5 to 1.7 μg/kg/day: thyroid-stimulating hormone (TSH), free thyroxine (FT4), high-sensitivity C-reactive protein (hs-CRP), interleukin 1 (IL-1), IL-6, IL-10, interferon gamma (INF-γ), tumor necrosis factor alpha (TNF-α), thiobarbituric acid-reactive substances (TBARS), activity of aminolevulinic acid dehydratase (δ-ALA-D), nonprotein and total thiol (NP-SH and T-SH) groups, total cholesterol (TC), high-density lipoprotein cholesterol (HDL-C), low-density lipoprotein cholesterol (LDL-C) and triglycerides (TG). Generalized estimating equation (GEE) modeling was used to analyze the effects of LRT (at pre-treatment, 6 months and 12 months) on those variables. The hypothyroidism status (i.e., overt or subclinical hypothyroidism) was included as a confounder in all analyses. An additional GEE post hoc analysis was made to compare time points.

**Results:**

There was a significant decrease in TSH over time (P < 0.0001), (initial levels were on average 32.4 μIU/mL and 10.5 μIU/mL at 12 months). There was a significant increase in FT4 (P < 0.0001) (initial levels were on average 0,8 ng/dL and 2.7 ng/dL at 12 months). There were significant changes in interleukin levels over time, with a significant increase in IL-10 (P < 0.0001) and significant decreases in IL-1 (P < 0.0001), IL-6 (P < 0.0001), INF-γ (P < 0.0001) and TNF-α (P < 0.0001). No significant difference in hs-CRP over time was observed (P < 0.284). There was a significant reduction in NP-SH (P < 0.0001).

**Conclusions:**

This study observed significant changes in the inflammatory profile in hypothyroid patients under treatment, with reduction of pro-inflammatory cytokines and elevation of anti-inflammatory cytokine. In these patients, a decrease in low-grade chronic inflammation may have clinical relevance due to the known connection between chronic inflammation, atherosclerosis and cardiovascular events.

## Background

The full or parcial deficiency of thyroid hormone action is called hypothyroidism, which can be either overt (OH) or subclinical (SCH). SCH is characterized by a serum thyrotropin (TSH) level above the upper reference limit in combination with a normal level of free thyroxine (FT4), while OH is characterized by elevated TSH, in combination with subnormal FT4 [1]. The most common cause of primary hypothyroidism is chronic autoimmune thyroiditis, a disease also known as Hashimoto’s thyroiditis [1]. It is characterized by diffuse infiltration of the gland with sensitized T lymphocytes, with gradual destruction and fibrous replacement of the thyroid parenchymal tissue, elevated serum antithyroid antibodies, evidence of goiter or thyroid glandular atrophy and dysfunction to varying degrees [2].

Hypothyroidism is a prevalent disorder [3–5], and both OH and SCH seem to exert deleterious effects on the cardiovascular system [6]. Several mechanisms may be involved in this interaction, and the increased risks of atherosclerosis and coronary heart disease are some of them [4, 7]. Atherosclerosis develops over a period of years; inflammation is implicated in all of its stages (from the initial leukocyte recruitment to eventual rupture of the unstable atherosclerotic plaque) and has also been considered the link between the traditional risk factors and evident modifications in the artery wall [8].

Numerous circulating inflammatory biomarkers are associated with increased acute coronary event risk. These biomarkers may reflect pathways involved in disease progression and may thus be potential tools for predicting atherosclerosis and cardiovascular events [9]. C-reactive protein (CRP) is one of the most widely studied biomarkers in the general population and has been used to assess cardiovascular risk in both healthy subjects and people with various disorders [10]. Most epidemiological evidence on the relevance of cytokines as inflammatory markers has been obtained for interleukin (IL)-6 [11, 12]. Long-term circulating IL-6 levels may be associated with coronary heart disease, such as the main risk factors already established [11]. A causal role for IL-6 signaling in coronary heart disease is still elusive, but has been considered [12]. IL-1, interferon gamma (IFN-γ) and tumor necrosis factor alpha (TNF-α) are also important both the development of coronary heart disease and plaque destabilization [13–15]. TNF-α levels are significantly higher in patients with myocardial infarction than in controls, and patients with persistently higher levels in the post-infarction period are at a three fold higher risk of developing new coronary episodes [15]. There is substantial evidence that IL-1 inhibition can reduce recurrent vascular events in a high-risk secondary prevention population [13]. High levels of systemic markers of IFN-γ activity predict long-term adverse prognosis in patients with stable angina pectoris, demonstrating the capacity to identify individuals with vulnerable lesions, despite their stable clinical conditions [14]. Circulating levels of several different pro-inflammatory cytokines in initially healthy people are associated with risk of coronary heart disease outcomes in an approximately log-linear manner [16].

Oxidative stress (OS) can also serve as both a monitor of inflammation and a pathophysiologic mediator of atherosclerosis [8, 17]. The association between hypothyroidism and increased OS is controversial, but greater OS has been observed in patients with SCH and OH in comparison with euthyroid controls [18–23]. Inflammation observed in patients with Hashimoto’s thyroiditis and hypothyroidism is considered the link between OS and increased risk of atherosclerosis and underlying cardiovascular disease in these patients [24–26].

Hypothyroidism is treated with substitutive doses of levothyroxine. Few studies have evaluated the effects of hypothyroidism treatment on either OS, with conflicting results [18–22], or systemic inflammation [19, 27–31], also with conflicting results. Thus, this study aims to assess inflammatory biomarkers and OS in patients with primary hypothyroidism at baseline and after 6 and 12 months of levothyroxine replacement therapy (LRT).

## Methods

### Subjects

Patients were recruited prospectively, in the outpatient endocrinology clinic, University Hospital of Santa Maria, Santa Maria, RS, Brazil. The study protocol was approved by the Human Ethics Committee of the Federal University of Santa Maria (number 0177.0.243.000.07). Before performing any procedure, all of the patients were extensively informed about the study and signed an informed consent form. Inclusion criteria were age between 18 and 69 years, recent OH defined as high thyrotropin (TSH greater than 4.2 μIU/mL) and low FT4 or SCH, defined as a high level of TSH with FT4 level within the reference range (0.93 to 1.71 ng/dl). Patients with SCH had been referred to the clinic because of presentation of goiter and its symptoms (dry skin, muscle weakness, impaired memory and thinking, fatigue, cold intolerance, constipation), with stable thyroid dysfunction for at least 6 months, without evidence of other recent or ongoing diseases.

Stringent exclusion criteria were defined to limit sources of confounding factors that could influence the clinical or laboratory parameters under investigation. We did not include patients with current treatment for thyroid dysfunction; diabetes mellitus; pregnancy; malignancy; active smoking; alcohol consumption; history of previous or recent respiratory disease; previous or recent cardiovascular disease; history of connective tissue diseases; or exposure to drugs such as interferon, amiodarone, lipid-lowering drugs, appetite suppressants, antioxidant vitamin supplementation, or beta-blockers.

A medical history, physical examination, thorax radiogram, spirometry, electrocardiogram, transthoracic Doppler echocardiogram and laboratory investigations were performed in all patients. Body mass index (BMI) was calculated by dividing the body weight by the square of the height (kg/m ^2^). The levothyroxine (FT4) replacement therapy was performed with an average dose of 1.5 to 1.7 μg/kg/day. SCH required a lower dose. Patients were instructed to take the medication orally, with water, always in a single dose in the morning, after fasting (between 30 and 60 min before the first meal). Inflammatory and oxidative stress profiles were performed simultaneously with the collection of the thyroid profile, at 6 and 12 months follow-up.

### Laboratory assessments

At baseline and after 6 and 12 months of LRT, blood samples were collected to assess thyroid profile (TSH, FT4), lipid profile (total cholesterol [TC], high-density lipoprotein cholesterol [HDL-C], low-density lipoprotein cholesterol [LDL-C], triglycerides [TG]) and inflammatory biomarkers (high-sensitivity CRP [hs-CRP], IL-1, IL-6, IL-10, INF-γ and TNF-α). In addition, we measured markers of OS (aminolevulinico acid dehydratase [δ-ALA-D], total thiol group [T-SH] and nonprotein thiol group [NP-SH] and thiobarbituric acid-reactive substances [TBARS]). Dosages of antithyroglobulin antibody (antiTG Ab) and antiperoxidase antibody (antiTPO Ab) were performed only at pre-treatment.

Blood samples were collected by venipuncture after fasting for 12 h. The samples were centrifuged for 15 min at 2500 xg and serum aliquots were stored at - 20 degrees centigrade to study IL-1, IL-6, IL-10, and INF-γ, TNF-α. An aliquot of whole blood was sent to the laboratory of Biochemistry of the Natural and Exact Sciences Center for further measures of δ-ALA-D activity and NP-SH, T- SH and TBARS levels.

The thyroid profile (TSH, FT4, antibodies) was measured by electrochemiluminescence (Immulite 2000, Siemens Healthcare Diagnostics, USA). We considered the following reference values: TSH: 0.27 to 4.2 uIU/mL, FT4: 0.93 to 1.71 ng/dl; antiTPO Ab: up to 35 IU/ml and antiTG Ab: up to 115 IU/ml.

The lipid profile was measured by the homogeneous enzyme method (Dimension 2008, Siemens Healthcare Diagnostics, USA). TC and TG were measured in serum according to the technique recommended by the manufacturer. HDL-C was measured in plasma supernatant after precipitation of lipoproteins containing apolipoprotein B with dextran sulfate and magnesium chloride as previously described by Bachorik and Albers [32].

LDL-C was estimated from the Friedewald equation [33]. Baseline values were: TC: up to 200 mg/dl, TG: 150 mg/dl, HDL-C: above 40 mg/dl for men and above 50 mg/dL for women; LDL-C: up to 130 mg/dl.

Hemoglobin was measured using the Sysmex automated system.

CRP was measured in serum by the immunoturbidimetric method, which measures hs-CRP (Dimension 2008, Siemens Healthcare Diagnostics, USA). The reference value was less than 0.5 mg/dl.

Cytokines were measured by enzyme-linked immunosorbent assay (ELISA) using commercial kits for human IL-1, IL-6, IL-10, INF-γ, and TNF-α (eBioscience, San Diego, USA) according to the manufacturer’s instructions.

Lipid peroxidation was determined by TBARS quantification using the method of Ohkawa et al. [34] with modifications. Red blood cells were isolated and proteins precipitated with 5 % trichloroacetic acid on ice for 30 min. The precipitate was removed by centrifugation at 3,000 rpm and the supernatant were then incubated at 100 °C for 60 min in an acidic medium containing 0.35 % phosphoric acid, and 0.28 % thiobarbituric acid. After centrifugation, the reaction product was determined at 532 nm using malondialdehyde as a standard. The results are expressed as nmol MDA/ml of erythrocytes.

Thiol oxidation was assessed by determination of T-SH and NP-SH, as described by Ellman [35]. For NP-SH level 300 microliters of isolated red blood cells were hemolyzed with 100 μL of 10 % Triton solution for 10 min. Following by protein fraction precipitation with 200 μL of 20 % trichloroacetic acid, the free SH groups (NP-SH) were determined in the supernatant using the colorimetric method performed in a potassium phosphate buffer (1 M, pH 7.4) with the presence of 10 mM of 5,,5′dithio(bis-nitrobenzoic acid) (DTNB). A standard curve was constructed using glutathione. The NP-SH level was measured at 412 nm and is expressed as nmol NP-SH/ml erythrocytes.

The T-SH level was determined in plasma and the colorimetric assay was performed in 0.85 ml of potassium phosphate buffer (0.3 M, pH 7.0), using 0.05 ml of 5,5′dithio(bis-nitrobenzoic acid). A standard curve was constructed using glutathione to determine the total thiol groups. The T-SH level was measured at 412 nm and is expressed as nmol T-SH/ml of plasma.

δ-ALA-D was determined in whole blood according to the method of Sassa [36]. Enzyme activity was measured in the presence or absence of the reducing agent dithiothreitol (DTT). The enzymatic reaction in hemolyzed blood (after mixing with Triton 0.05 %) was started after 10 min of sample pre-incubation, by adding the substrate aminolevulinic acid (ALA), followed by incubation at 37 °C for 60 min. The reaction product (porphobilinogen) was determined using Ehrlich’s reagent, which reacted and formed a pink-colored product measured at 555 nm. The activity is expressed as nmol PBG/hour/ml of blood.

### Statistical analysis

Data were analyzed with SPSS 18.0 (SPSS Inc. Chicago, IL, USA). Generalized estimating equation (GEE) modeling was used to analyze the effects of LRT (at pre-treatment, 6 months, and 12 months) on thyroid and lipid profiles, and on systemic inflammation and OS. The type of model and the link function were defined according to dependent variable distribution. Each parameter was analyzed as to its nature to choose the most suitable working correlation matrix. The model was chosen as the criterion of lowest quasi-likelihood under the independence model criteria (QIC). The hypothyroidism status (i.e., OH or SCH) was included as a confounder in all analyses.

Because 17 models were tested, we considered P-values less than 0.0025 (α_Bonf_ = 0.05/17) to be significant. Eight models were significant at the 0.0025 level and underwent an additional GEE post hoc analysis to compare time points. In these analyses, corrected *P*-values were calculated, and alpha was set at 0.05. Data are expressed as means and standard deviations, means and 95 % confidence intervals (95 % CIs) or numbers (n) and percentages (%), as indicated in the table footnotes.

## Results

Clinical, anthropometric, and baseline thyroid profiles of the study participants are given in Table 1. The group included 17 patients with high TSH, 11 of whom had diminished FT4 (OH) and 6 of whom had normal FT4 (SCH). The initial 17 patients, 16 were reassessed at 6 and 12 months of treatment with levothyroxine. Thyroid, lipid, hemoglobin, inflammatory and OS profiles of hypothyroid patients at pre-treatment and 6 and 12 months after LRT are shown in Table 2. All patients had increased anti-thyroglobulin antibodies and/or antibody antiperoxidase levels.

**Table 1:** Clinical, anthropometric and pre-treatment thyroid profile of the study

Variables	Hypothyroidism (TSH ↑) (n = 17)
Gender (males)	6 (35.3)
Age (years)	45.8 (15.2)
BMI (Kg/m^2^)	28.4 (5.8)
TSH (ulU/ml)	32.1 (30.2)
FT4 (ng/dl)	0.8 (0.4)
AntiTG Ab (IU/mL)	979.9 (1386.0)
AntiTPO Ab (IU/mL)	398.0 (309.9)

Data are mean and (standard deviation) or n and (%) for gender

**Table 2:** Generalized estimating equation (GEE) models to evaluate the effects of Levothyroxine replacement therapy (LRT) on inflammatory, oxidative stress, hemoglobin, and lipid profile parameters of hypothyroid patients

Parameters	Pre-treatment	Six months	Twelve months	Wald Chi-square	df	*P*-value
	Mean (95 % CI)	Mean (95 % CI)	Mean (95 % CI)			
TSH (μIU/mL)	32.4 (18.0–30.4)	20.7 (8.6–49.8)	10.5 (6.3–17.4)	18.496	2	<0.0001
FT4 (ng/dL)	0.8 (0.7–0.9)	3.4 (1.8–6.6)	2.7 (1.3–5.8)	33.684	2	<0.0001
IL-10 (pg/mL)	132.7 (124.5–141.0)	160.7 (150.7–170.6)	184.6 (174.8–194.4)	181.076	2	<0.0001
IL-1 (pg/mL)	73.0 (68.5–77.4)	57.9 (52.6–63.1)	45.0 (40.4–49.6)	321.214	2	<0.0001
IL-6 (pg/mL)	90.1 (83.7–96.4)	70.5 (63.9–77.1)	49.9 (44.1–55.6)	512.104	2	<0.0001
INF-γ (pg/mL)	144.8 (141.0–148.6)	98.6 (93.1–104.5)	80.0 (75.8–84.3)	386.001	2	<0.0001
TNF-α (pg/mL)	122.8 (120.5–125.1)	90.4 (86.8–94.0)	66.2 (61.6–71.1)	341.402	2	<0.0001
hs-CRP (mg/dl)	0.6 (0.3–1.1)	0.5 (0.3–0.8)	0.9 (0.5–1.7)	2.519	2	0.284
NP-SH (nmol NP - SH/mL erit)	1522.4 (1374.9–1670.0)	1098.1 (929.3–1267.0)	1004.6 (757.5–1251.7)	23.528	2	<0.0001
Total-SH (nmol T -SH/ml plasma)	823.3 (683.5–963.2)	624.0 (537.1–710.8)	660.5 (584.2–772.8)	5.388	2	0.068
δ-ALA-D (nmol PBG/hour/ml of blood)	3.6 (2.7–4.4)	3.1 (1.9–4.3)	3.0 (2.1–4.0)	0.644	2	0.725
TBARS (nmol MDA/mL erit)	5.3 (4.0–6.7)	7.2 (4.8–8.5)	6.7 (4.8–8.5)	2.862	2	0.239
Hemoglobin (g/dL)	13.1 (12.6–13.6)	13.4 (13.0–13.9)	13.6 (13.2–14.0)	8.252	2	0.016
TC (mg/dL)	201.5 (176.7–244.2)	198.4 (168.5–228.4)	198.4 (171.9–225.0)	1.567	2	0.457
HDL- C (mg/dL)	54.3 (48.8–59.7)	50.9 (41.8–60.0)	52.1 (45.8–58.4)	0.670	2	0.715
LDL- C (mg/dL)	124.2 (102.6–150.5)	115.5 995.5–139.7)	115.5 (99.0–134.7)	2.146	2	0.342
TG (mg/dL)	117.5 (88.1–156.9)	139.2 (99.2–195.4)	119.6 (85.3–167.7)	1.085	2	0.581

df = degrees of freedom; adjusted for overt hypothyroidism and subclinical hypothyroidism

**Fig. 1:**
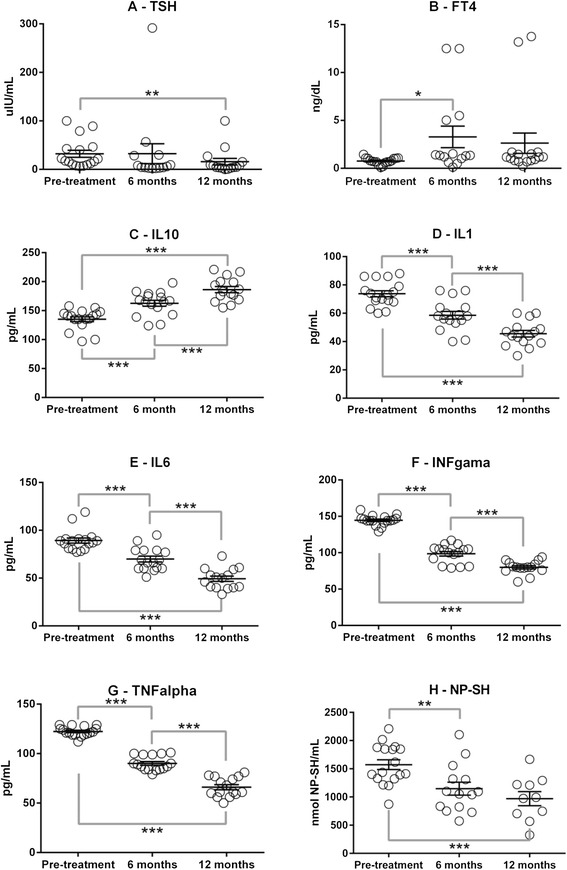
Post Hoc comparisons from significant generalized estimating equation. (GEE) models: Panel **A** (TSH); Panel **B** (FT4); Panel **C** (IL-10); Panel **D** (IL-1); Panel **E** (IL-6); Panel **F** (INF-γ); Panel **G** (TNF-α)and Panel H (NP-SH), as described in Table 2. *P ≤ 0.050; **P ≤ 0.010; ***P ≤ 0.001

GEE evaluation (Table 2) revealed a significant decrease in TSH over time (P < 0.0001). The initial TSH levels were on average 32.4 uIU/mL and the levels achieved at 12 months were 10.5 uIU/mL. In the post hoc analysis, significance was observed only at 12 months of LRT compared with pre-treatment (Fig. 1a). There was also significant modification in FT4 over time (P < 0.0001). The initial FT4 levels were on average 0.8 ng/dl and the levels achieved at 12 months were on average 2.7 ng/dl. In the post hoc analysis, a significant difference was noted between 6-months FT4 and pre-treatment (Fig. 1b).

Regarding inflammatory profile, there were significant changes in interleukin levels over time, with a significant increase in IL-10 (P < 0.0001) and significant decreases of IL-1 (P < 0.0001), IL-6 (P < 0.0001), INF-γ (P < 0.0001) and TNF-α (P < 0.0001). The GEE post hoc analyses revealed that the 6- and 12-month values differed significantly from pre-treatment (Fig. 1c to 1g). No significant difference in hs-CRP over time was noted (P = 0.284).

The evaluation of OS during follow-up showed significant reduction of NP-SH (P < 0.0001). In the post hoc analysis, 6 and 12-month NP-SH levels differed from pre-treatment (Fig. 1h). No significant change were observed in T-SH (P = 0.068), δ-ALA-D (P = 0.725) or TBARS (P = 0.239) levels over time.

An increasing trend was detected for hemoglobin levels (P = 0.016). There were no significant changes in TC (P = 0.457), HDL-C (P = 0.715), LDL-C (P = 0.342) or TG (P = 0.581) during follow-up.

## Discussion

The main finding of this open label study was that patients with hypothyroidism using levothyroxine presented significant changes in the inflammatory profile, with reduction of pro-inflammatory cytokines and increase of one anti-inflammatory cytokine. It was also observed reduction of one OS biomarker, although there were no significant changes in CRP or lipid profile.

Christ-Crain et al. [27] first reported increased CRP in patients with OH or SCH citing it as an additional risk factor for development of coronary heart disease in those patients, but further studies have shown conflicting results. Some studies have detected elevated CRP in individuals with hypothyroidism [5, 19, 24–26, 29, 37–39], while others have not [30, 40, 41]. Regarding the effect of LRT on CRP, in the current study, in accordance with the first report of Christ-Crain et al. [27] and others [30, 31, 37, 39, 41], the CRP level was unaffected. However, other studies have shown that normalization of thyroid state by LRT seems to effectively reduce serum CRP [19, 29, 38]. Marfella et al. [26] observed a reduction in CRP in treated SCH patients, but CRP was still significantly increased compared to the control group.

While pro-inflammatory cytokines have a detrimental role in atherosclerosis [8], the role of cytokines in Hashimoto’s thyroiditis is complex and often contradictory: a regulatory cytokine may either favor induction of tolerance against thyroid autoimmune disease or favor activation and/or exacerbation of autoimmune responses [2]. Drugarin et al. [42] observed increased serum IL-2, TNF-α and IFN-γ in subjects with OH due to autoimmune thyroiditis compared with healthy controls, in whom these cytokines were barely detectable. Recently, in patients with autoimmune thyroiditis, increased IL-6 and IL-15 (another pro-inflammatory cytokine) were detected, likely due to increased proliferation and increased pro-inflammatory cytokine synthesis in T-helper 17 cells [43]. Díez et al. [44] confirmed the relevance of activation of the TNF-α system in patients with hypothyroidism, showing that serum TNF-α and receptors of TNF-α were significantly higher than those detected in the control group. Thyroid hormone levels have been suggested to influence cytokine production. IL-6 level might be correlated with hypothyroidism severity because serum IL-6 level has been positively correlated with LRT dose and negatively correlated with FT4 level in hypothyroidism due to autoimmune thyroiditis [45]. Díez et al. [44] found no differences in TNF-α or receptors of TNF-α between patients with autoimmune and non-autoimmune hypothyroidism, implicating thyroid hormone deficiency per se in systemic cytokine production. Karanikas et al. [46] demonstrated no influence of thyroid hormone on cytokine production patterns by T cells, as they reported that patients with high titers of anti-TPO antibodies had significantly higher percentages of cells producing INF-γ and TNF-α than healthy controls.

Based on previous studies, an association between hypothyroidism and low-grade inflammation may be suggested. Taddei et al. [24] demonstrated a higher plasma IL-6 level, impaired endothelium dysfunction vasodilatation and reduced nitric oxide availability in patients with SCH compared with euthyroid controls. Türemen et al. [25] observed endothelial dysfunction and significantly higher serum IL-6 and TNF-α levels in SCH patients with autoimmune thyroiditis compared to controls. Patients with untreated SCH who underwent thromboendarterectomy due to asymptomatic severe internal carotid artery stenosis presented higher plasma TNF-α, IL-6, and interleukin-18 levels, and their atherosclerotic plaques presented an active inflammatory reaction with higher TNF-α levels and other characteristics of instability compared with non-SCH controls [26].

Regarding potential changes in cytokine levels with LRT, we found that one year of LRT produced significant reductions in pro-inflammatory cytokines (IL-1, IL-6, INF-γ, TNF-α) and an increase in IL-10. There are few data in the literature about the effect of levothyroxine on serum cytokines. Díez et al. [44] observed no reduction of the high levels of TNF-α and TNF-α receptors after normalization of thyroid function in hypothyroid patients. Guclu et al. [47] observed a significant decrease in serum IL-12 (one of the most important cytokines responsible for Th1-type cytokine responses), a statistically non-significant decrease in IFN-γ and no change in serum IL-2 or IL-4 in patients with hypothyroidism due to Hashimoto’s thyroiditis who underwent 12 weeks of LRT. Marfella et al. [26] observed significantly lower plasma TNF-α, IL-6 and IL-18 levels, low pro-inflammatory cytokine levels and macrophage infiltration in atherosclerotic lesions of treated SCH patients compared to untreated SCH patients.

The association between hypothyroidism and increased OS is controversial, as is the effect of LRT on oxidative stress biomarkers. In the current study, NP-SH was significantly reduced by 12 months of LRT, but no significant changes were observed in T-SH, δ-ALA-D or TBARS. Some studies have shown reduction of oxidative stress after LRT, [18, 23, 29], while other studies have reported no modification [19, 20]. Marfella et al. [26] detected elevated OS biomarkers in atherosclerotic plaques of SCH subjects compared to control plaques, and higher levels of OS in plaques of untreated SCH patients in comparison with plaques of treated SCH patients. The restoration of endothelial function by systemic administration of indomethacin (cyclooxygenase inhibitor) or local infusion of vitamin C (an antioxidant) reinforces that OS may be a link between the inflammation and endothelial dysfunction observed in SCH patients [24]. According to Marfella et al. [26], OS reduction can play an important role in reducing the inflammatory activity in atherosclerotic plaques observed in patients with SCH treated with thyroxine.

In our sample, which included both OH and SCH patients, there were no significant changes in TC, HDL, LDL or TG over 12 months of LRT. Dyslipidemia is a common finding in patients with OH, with elevated TC and LDL [3, 27, 48], which usually improve with levothyroxine use [48]. Less clear is the relationship between SCH and lipid profile. Some studies have shown no alterations in lipid profile in SCH patients compared with controls [4, 27, 40, 41], while others have observed abnormalities in serum cholesterol or TG in SCH [5, 38]. Teixeira et al. [49] have suggested that SCH presents an intermediary lipid profile between that observed in normal individuals and that usually observed in OH. The role of levothyroxine in changing the lipid profile in SCH is also not clear. Iqbal et al. [50], in a large epidemiological study, and Razvi et al. [51], in a study of 100 patients with SCH, reported TC and LDL-C reductions with LRT. Teixeira et al. [49] observed a significant lipid profile improvement after 1 year of LRT. However, other studies did not report modifications of lipid levels with LRT [19, 29, 31].

Three older meta-analyses concluded that thyroxine substitution has no effect on TG and HDL-C [48, 52, 53], whereas TC and LDL-C levels were reduced, with the most pronounced effect in those with the highest pretreatment serum TSH [53] and serum TC [48, 52]. However, a more recent meta-analysis based on the Cochrane methodology, including 12 randomized controlled trials of 6–14 months’ duration, reported that the beneficial effects of treatment were slight and that only TC was affected [54].

Knowledge about the effects of levothyroxine on inflammatory, OS and lipid profiles has been increasing. The results are conflicting, but there are growing indications of favorable effects of levothyroxine on adhesion molecule levels in SCH [55], impaired endothelial function and hemodynamic profile both in OH and SCH [28, 37, 41, 51, 56]. There are also indications that young patients with treated SCH, in comparison with untreated SCH patients, present a significantly lower risk of developing heart failure [57], a significantly lower all-cause mortality [58] and fewer events of ischemic heart disease [59]. The decreased immune overactivity observed in our study may be a mechanism contributing to these previous results.

Our study has strengths and limitations. The strengths are its prospective nature, the number of investigated inflammatory biomarkers, the rigorous patient selection and the careful statistical analysis with correction for confounding factors. The main limitations are the rather small sample size, heterogenous sample (composed of both OH and SCH), lack of a control group and lack of a placebo-controlled group.

## Conclusion

In conclusion, this study observed a significant reduction of pro-inflammatory cytokines and an increase of one anti-inflammatory cytokine in hypothyroid patients using levothyroxine. In these patients, the reduction of low-grade chronic inflammation may have clinical relevance because of the known connection between chronic inflammation, atherosclerosis and cardiovascular events.

## Abbreviations

δ-ALA-D: Aminolevulinic acid dehydratase
antiTG Ab: antithyroglobulin antibody
antiTPO Ab: antiperoxidase antibody
BMI: Body mass index
CRP: C-reactive protein
FT4: Free thyroxine
HDL-C: High-density lipoprotein cholesterol
hs-CRP: high-sensitivity C-reactive protein
IL-1: Interleukin 1
IL-6: Interleukin 6
IL-10: Interleukin 10
INF-γ: Interferon gamma
LRT: Levothyroxine replacement therapy
LDL-C: Low-density lipoprotein cholesterol
NP-SH: Nonprotein thiol group
OH: Overt hypothyroidism
OS: Oxidative stress
SCH: Subclinical hypothyroidism
TBARS: Thiobarbituric acid-reactive substances
TSH: Thyroid-stimulating hormone, TC, Total cholesterol
T-SH: Total thiol group
TG: Triglycerides
TNF-α: Tumor necrosis factor alpha
